# Complete mitochondrial genome of Asian longhorned tick, *Haemaphysalis longicornis*, Neumann, 1901 (Acari: Ixodida: Ixodidae) identified in the United States

**DOI:** 10.1080/23802359.2021.1922100

**Published:** 2021-07-19

**Authors:** Ji-Yeon Hyeon, Holly McGinnis, Maureen Sims, Zeinab H. Helal, Junwon Kim, David H. Chung, Guillermo R. Risatti, Dong-Hun Lee

**Affiliations:** a Connecticut Veterinary Medical Diagnostic Laboratory, University of Connecticut, Storrs, CT, USA; b Department of Pathobiology and Veterinary Science, College of Agriculture, Health and Natural Resources, University of Connecticut, Storrs, CT, USA

**Keywords:** Tick, *Haemaphysalis longicornis*, mitogenome, phylogenetic analysis

## Abstract

*Haemaphysalis longicornis* (Ixodida: Ixodidae), the Asian longhorned tick, which is native to temperate East Asia, has been recently detected in the northeastern region of the United States, drawing concerns about its potential impact on the US animal and public health sectors. Knowledge about the genetic features of *H. longicornis* found in the US is limited. Therefore, we sequenced the complete mitochondrial genome (mt-genome) from two *H. longicornis* ticks recently collected in the State of New York, USA, in 2020. These ticks were morphologically identified and tested for tick-borne pathogens at the Connecticut Veterinary Medical Diagnostic Laboratory (Storrs, CT). The mt-genome was 14,694 bp in length and encoded 37 genes, including 13 protein-coding genes, 22 transfer RNAs, and two ribosomal RNAs. Phylogenetic analysis showed that the mt-genome clustered with those of other *H. longicornis* identified in China. The mt-genome sequence was 99.7% identical to a *H. longicornis* mt-genome (GenBank: MK439888) collected in China. The *cox1* gene haplotype in these ticks belonged to the H1 type, which is the dominant haplotype present in central NJ and Staten Island, NY. The complete mt-genome data are needed to provide insights into genetic changes and phylogenetic studies of *H. longicornis* ticks.

In Asia, *Haemaphysalis longicornis* Neumann (Ixodida: Ixodidae), the Asian longhorned tick, is considered a primary vector for the Severe Fever with Thrombocytopenia Syndrome virus (SFTSV) (Liu et al. 2014). These ticks also are competent vectors for the Spotted Fever Group *Rickettsia* spp (Zou et al. 2011) and *Rickettsia japonica* causing Japanese spotted fever, a fetal zoonotic disease (Noguchi et al. 2018). In addition, other tick-borne pathogens including *Anaplasma*, *Borrelia*, and *Ehrlichia* species have also been detected in *H. longicornis* (Sun et al. 2008; Egizi et al. 2020). While *H. longicornis* is indigenous to East Asia and southeast Russia (Beard et al. 2018; Egizi et al. 2020), it has also been found in the U.S. It was initially identified in 2017 on a sheep in New Jersey, and since then, the species has been reported in 15 states. Recent reports of heavy *H. longicornis* infestations on cattle (*Bos taurus*) and white-tailed deer (*Odocoileus virginianus*) together with wide distribution of this invasive species have drawn concern about its potential impact on US livestock and public health sectors (Egizi et al. 2020). Considering its importance, there is limited knowledge on the genetic features of *H. longicornis* found in the U.S.

Mitochondrial (mt)-genomes are characterized by their simple structure, small molecular weight, maternal inheritance, relatively high mutation rates, and the lack of recombination (Wang et al. 2019a, 2019b). With these properties, molecular approach using mitochondrial genome (mt-genome) has been recently used for identification and characterization of ticks as well as molecular evolution, phylogeny, and genealogy of ticks (Liu et al. 2013, 2020; Cameron 2014; Geng et al. 2017; Wang et al. 2019a, 2019b; Egizi et al. 2020). As of 11 February 2021, only five complete mt-genome sequences have been reported in NCBI GenBank database, mainly originated from China. In the present study, we report the first complete mt-genome sequence of two *H. longicornis* ticks found during the end of June in 2020 in the state of New York, U.S.

In June 2020, two tick specimens collected from Rockland County, NY (latitude 41.147594, and longitude −73.989304) were submitted to the Connecticut Veterinary Medical Diagnostic Laboratory (CVMDL, Holly McGinnis, holly.mcginnis@uconn.edu, Maureen Sims, maureen.sims@uconn.edu), University of Connecticut for identification (speciation) and testing for tick-borne pathogens. The tick 1 (20-2319) was identified as an adult female *H. longicornis* tick and the tick 2 (20-2320) as a nymph stage *H. longicornis* using the morphological key of previous study (Egizi et al. 2019). These moderately engorged ticks were tested by SYBR green based qPCR for tick-borne pathogens (Pietila et al. 2000; Adelson et al. 2004; Pesquera et al. 2015; Muhammad et al. 2019). The tick 2 was positive for *Anaplasma phagocytophilum.* DNA was extracted from ticks using a Nucleospin Tissue kit (Macherey-Nagel, Bethlehem, PA), and genome sequencing was performed on MiSeq sequencer (Illumina, San Diego, CA). Quality-filtered reads (*Q* > 30 and minimum length >100) by BBduk (https://sourceforge.net/projects/bbmap) were mapped to the reference sequence (GenBank accession no. MK450606) using Bowtie 2 (Langmead and Salzberg 2012). Consensus sequences were annotated by MITOS (Bernt et al. 2013).

The complete mt-genome sequences of all *Haemaphysalis* ticks were retrieved from the GenBank for phylogenetic analysis (*n* = 22). The sequences were aligned using MAFFT (https://mafft.cbrc.jp/alignment/software/). The maximum-likelihood (ML) phylogeny was constructed using RAxML-v8 (Stamatakis 2014) using the GTR + G4 substitution model with 1000 rapid bootstrapping. We identified the *cox1* haplotype of two samples. Egizi et al. (2020) showed that three *cox1* haplotypes (H1–3) of *H. longicornis* were detected within the U.S. A minimum spanning haplotype network was created using PopART 1.7 (Leigh and Bryant 2015) with eight randomly selected *cox1* sequences of each haplotype (Egizi et al. 2020).

The mt-genome sequences of two ticks obtained in this study were identical. The mt-genome was 14,694 bp in length and classified as type III mt-genome according to the previous study (Montagna et al. 2012). It encoded 13 protein-coding genes (PCGs) (*cox1–3, nd1–6, nd4L, cob, atp6, and atp8*), 22 tRNA genes, two rRNA genes including 16S rRNA (1205 bp) and 12S rRNA (767 bp), and two noncoding regions (NCRs) located at *rrnS*-*trnI* and *trnL1*-*trnC*. A tandem repeat was found in *apt8* gene using Tandem Repeat Finder (Benson 1999). The nucleic acid base content was 38.3% A, 13.0% C, 9.7% G, and 39.0% T. ATT start codon was used by *nd1*, *nd2*, *nd3*, *nd4L*, *nd5*, *cox1*, and *cox2*. ATG codon was used by *atp6*, *cob*, and *nd4*, ATA codon by *atp8* and *cox3*, and ATC codon by *nd6*. The most PCGs were terminated by TAA stop codon except *nd3* and *cob* that use TAG and *nd6* uses the single T, as the stop codon. Among the 37 genes, nine PCGs and 14 tRNAs were located on the forward strand (H-strand), while the remaining genes were transcribed on the reverse strand (L-strand).

The phylogenetic analysis showed that the *H. longicornis* mt-genome formed a monophyletic cluster with other *H. longicornis* which is well-supported by high bootstrap value (Figure 1(a)). The mt-genome sequence was 99.7% identical to a *H. longicornis* collected in China on 20 March 2018 (GenBank accession no. MK439888). The minimum spanning tree showed that the mt-genome sequences belong to the H1 haplotype which is the dominant in central New Jersey and Staten Island, New York (Figure 1(b)).

**Figure 1.**
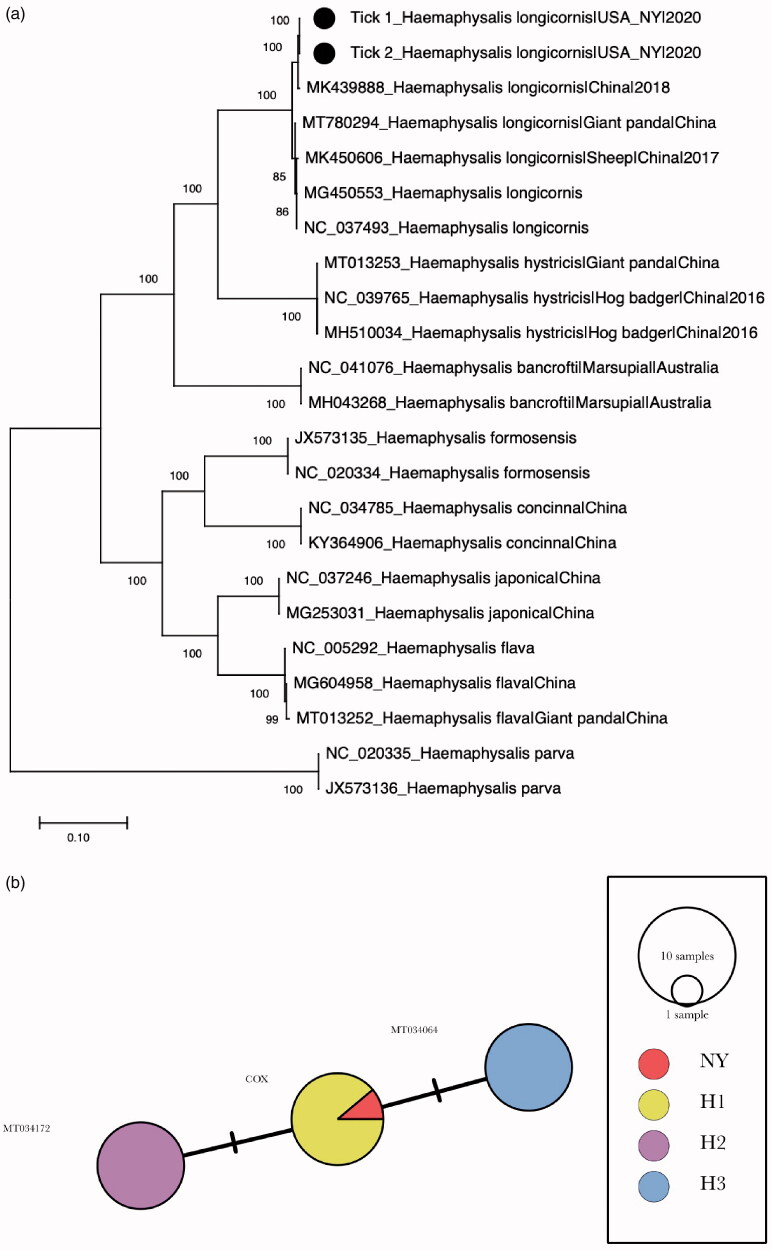
Phylogenetic analysis of the complete mt-genome *Haemaphysalis longicornis* of this study. (a) Maximum-likelihood phylogeny of complete *H. longicornis* mt-genomes. The percentages of the bootstrap test in 1000 replicates were shown above the branches. The *H. longicornis* mt-genome sequenced this study are indicated with black circles. Scale bar indicates nucleotide substitutions per site. (b) Minimum spanning network of *H. longicornis cox1* haplotypes identified in the United States. Eight *H. longicornis cox1* sequences per haplotype occurring in the United States (H1, H2, and H3) and the *H. longicornis cox1* sequence of this study are included in the analysis.

The widespread and establishment of this invasive species in the U.S. highlights the risk to public health since the invasive ticks are capable of transmitting pathogens of human and veterinary concern. The complete mt-genome of *H. longicornis* found in the U.S. would provide important information to understand genetic diversity and epidemiology of this invasive tick species.

## Data Availability

The genome sequence data that support the findings of this study are available in GenBank of NCBI at https://www.ncbi.nlm.nih.gov/ under the accession no. MW602986. The associated BioProject number is PRJNA705377, and Bio-Sample numbers are SAMN18086111 and SAMN18086112. The NGS library are stored at the CVMDL, University of Connecticut.
